# Uniform Hospital Accounts

**Published:** 1892-01-30

**Authors:** 


### Uniform Hospital Accounts.

### IMPORTANT ACTION OF METROPOLITAN HOSPITAL SUNDAY FUND AND THE SECRETARIES.

Ever since the Hospital Conference in 1883, when the subject was first dealt with in a practical form, there has been a movement in favour of the establishment of one uniform system of Hospital Accounts. Without it anomalies must prevail, and anything like a definite and just comparison of the expenses of working at one hospital as compared with another has been difficult, if not impossible. Early in 1891 the Distribution Committee of the Hospital Sunday Fund circulated a form of accounts, proposed by a recognised authority, which resulted in the formation of a Committee of Hospital Secretaries, which held its first meeting at the Hotel Metropole on March 9th, 1891. An interim report was presented in June of the same year, but as it was thought desirable to prepare an index of classification on the plan of Mr. Michelli's Glossary, published in *The Hospital Annual*, this laborious undertaking was cheerfully entered upon and brought to a successful conclusion in December, when it was submitted to the Distribution Committee of the Hospital Sunday Fund, together with the form of accounts. The Committee of Distribution considered the various alterations to be improvements acquiesced in them without reserve, and determined to recommend their adoption as the model for the return which the Fund requires the hospitals to fill up every year. As it was considered desirable that they should receive the approval of the secretaries of all the hospitals receiving grants from the Hospital Sunday Fund, a meeting was held on the 18th inst., at the Hotel Metropole, Mr. B. Burford Rawlings in the chair, when the following resolutions were adopted :—

On the motion of Mr. Thomas Ryan, Secretary, St. Mary's Hospital, W., seconded by Mr. Arthur E. Reade, Secretary, Charing Cross Hospital: "That this special general meeting of secretaries of metropolitan hospitals convened to consider a form of income and expenditure account originally drafted by the Council of the Hospital Sunday Fund, formulated by a Committee of Hospital Secretaries specially appointed for the purpose, and adopted by the Distribution Committee of the Hospital Sunday Fund, hereby approves of the said form of account, and considers the same suitable for uniform adoption by the hospitals of London for publication in their annual reports; and the secretaries present undertake to use their influence with the committees of their respective institutions with the view of securing the adoption of the form by them."

On the motion of Mr. Newton H. Nixon, Secretary, University College Hospital, seconded by Mr. Adrian Hope, Secretary, Hospital for Sick Children, Great Ormond Street, W.C.: "That a copy of the form of account and of the foregoing resolution be sent, through the Secretary, to the committee of every hospital receiving a grant from the Hospital Sunday Fund, accompanied by the suggestion that, in the interest of uniformity of hospital accounts, they would adopt it as the form for publication in their annual report, and with a request that they would kindly desire their Secretary to communicate their decision to our committee."

On the motion of Mr. Conrad W. Thies, Secretary, Royal Free Hospital, seconded by Mr. G. A. Cross, Secretary, London Homœopathic Hospital: "That the index of classification, with the accompanying synopsis, be adopted."

On the motion of Mr. F. Clare-Melhado, Secretary, Middlesex Hospital, seconded by Mr. R. A. Owthwaite, Secretary, City of London Lying-in Hospital: "That a Committee of Reference be appointed, to whom the secretaries may refer any difficulties they may experience in connection with the new form of account, and to whom also the committee of the various hospitals may communicate their decisions respecting its adoption."

The Committee submitted a statement of expenditure incurred by them in the conduct of this business, which, it is reasonable to hope, will be readily refunded by the Council of the Metropolitan Hospital Sunday Fund.

The resolutions speak for themselves, and will, we believe, commend themselves to the approval not only of the Hospital Committees, of the subscribers and the general public, but of the authorities of the provincial, Scotch, and Irish hospitals also. If so, the importance and value of this new departure cannot be over-estimated. Much praise is due to Mr. Burford Rawlings, Mr. Michelli, Mr. Owthwaite, Mr. Thies, and Mr. Ryan, all of whom have worked hard and done good service, as well as many other secretaries. The form of accounts is in the main that which was originally introduced at the Queen's Hospital, Birmingham, so long ago as 1869, and subsequently at the Seamen's Hospital, Greenwich; St. Mary's Hospital, Paddington, and elsewhere. Mr. Thomas Ryan has displayed commendable energy in connection with this work, and deserves praise for the enthusiasm and self-denial he has displayed in its progress. The form of accounts as finally approved will be found on the next page, and a list of the necessary account books, with specimen pages of each, will be found in *The Hospital Annual*, published at The Hospital office, 140, Strand, W.C.

.................................................*Hospital.*

**Dr.** INCOME AND EXPENDITURE ACCOUNT for the year ending the 31st December, 189 **Cr.**

As settled by the Committee of Hospital Secretaries and the Distribution Committee of Hospital Sunday Fund.

| INCOME. | £ | s. | d. | £ | s. | d. | £ | s. | d. |
|---|---|---|---|---|---|---|---|---|---|
| A. ORDINARY— | | | | | | | | | |
| I. ANNUAL SUBSCRIPTIONS (*see page* ) ... ... | | | | | | | | | |
| II. DONATIONS (*see page* ) | | | | | | | | | |
| Boxes (*see page* ) | | | | | | | | | |
| III. HOSPITAL SUNDAY FUND | | | | | | | | | |
| IV. HOSPITAL SATURDAY FUND | | | | | | | | | |
| V. CONGREGATIONAL COLLECTIONS (apart from Hospital Sunday Fund) | | | | | | | | | |
| VI. ENTERTAINMENTS ... | | | | | | | | | |
| VII. INVESTED PROPERTY— | | | | | | | | | |
| Dividends... ... ... | | | | | | | | | |
| Income Tax Returned ... | | | | | | | | | |
| Interest on Deposit Account ... ... ... | | | | | | | | | |
| Rents ... ... ... | | | | | | | | | |
| VIII. NURSING INSTITUTION— | | | | | | | | | |
| Private Nurses ... ... | | | | | | | | | |
| Nurses' and Probationers' Fees ... ... ... | | | | | | | | | |
| IX. PATIENTS' PAYMENTS— | | | | | | | | | |
| In-Patients ... | | | | | | | | | |
| Out-Patients ... ... | | | | | | | | | |
| X. OTHER RECEIPTS ... | | | | | | | | | |
| TOTAL ORDINARY INCOME | | | | | | | | | |
| B. EXTRAORDINARY— | | | | | | | | | |
| LEGACIES— | | | | | | | | | |
| The Executors of | | | | | | | | | |
| TOTAL EXTRAORDINARY INCOME | | | | | | | | | |
| | | | | | | | £ | | |

| EXPENDITURE. | £ | s. | d. | £ | s. | d. | £ | s. | d. |
|---|---|---|---|---|---|---|---|---|---|
| A. MAINTENANCE— | | | | | | | | | |
| I. PROVISIONS— | | | | | | | | | |
| Meat ... ... | | | | | | | | | |
| Fish, Poultry, &c. ... | | | | | | | | | |
| Butter, Cheese, &c. ... | | | | | | | | | |
| Eggs ... ... ... | | | | | | | | | |
| Milk ... ... ... | | | | | | | | | |
| Bread, Flour, &c. ... | | | | | | | | | |
| Grocery ... ... ... | | | | | | | | | |
| Vegetables ... ... | | | | | | | | | |
| Malt Liquors ... ... | | | | | | | | | |
| II. SURGERY & DISPENSARY— | | | | | | | | | |
| Drugs, Chemicals, Disinfectants, &c... ... | | | | | | | | | |
| Dressings, Bandages, &c. | | | | | | | | | |
| Instruments & Appliances | | | | | | | | | |
| Ice and Mineral Waters . | | | | | | | | | |
| Wine and Spirits | | | | | | | | | |
| Sundries ... ... | | | | | | | | | |
| III. DOMESTIC— | | | | | | | | | |
| Renewal of Furniture ... | | | | | | | | | |
| Bedding and Linen | | | | | | | | | |
| Hardware, Crockery, Brushes, &c. ... | | | | | | | | | |
| Washing ... ... ... | | | | | | | | | |
| Cleaning and Chandlery . | | | | | | | | | |
| Water ... ... ... | | | | | | | | | |
| Fuel and Lighting ... | | | | | | | | | |
| Uniforms ... ... | | | | | | | | | |
| Sundries ... ... ... | | | | | | | | | |
| IV. ESTABLISHMENT CHARGES— | | | | | | | | | |
| Rates and Taxes... ... | | | | | | | | | |
| Insurance ... ... ... | | | | | | | | | |
| Garden ... ... ... | | | | | | | | | |
| Annual Cleaning... ... | | | | | | | | | |
| Repairs (Ordinary) | | | | | | | | | |
| V. RENT ... ... | | | | | | | | | |
| VI. SALARIES, WAGES, &c.— | | | | | | | | | |
| Medical ... ... ... | | | | | | | | | |
| Dispensing ... ... | | | | | | | | | |
| Nursing ... ... ... | | | | | | | | | |
| Other Salaries and Wages | | | | | | | | | |
| Pensions ... ... ... | | | | | | | | | |
| VII. MISCELLANEOUS— | | | | | | | | | |
| Printing, Stationery, Postage, and Advertisements ... ... | | | | | | | | | |
| Sundries ... ... ... | | | | | | | | | |
| B. ADMINISTRATION— | | | | | | | | | |
| I. MANAGEMENT— | | | | | | | | | |
| Official Salaries ... ... | | | | | | | | | |
| Commission ... ... | | | | | | | | | |
| Pensions ... ... ... | | | | | | | | | |
| Official Printing and Stationery ... ... | | | | | | | | | |
| Official Postage and Telegrams ... ... | | | | | | | | | |
| Official Advertisements... | | | | | | | | | |
| Law Charges ... ... | | | | | | | | | |
| Interest on Loan... ... | | | | | | | | | |
| Auditors' Fee ... ... | | | | | | | | | |
| Sundries ... ... ... | | | | | | | | | |
| II. FINANCE— | | | | | | | | | |
| Appeals ... ... ... | | | | | | | | | |
| Festival ... ... ... | | | | | | | | | |
| TOTAL ORDINARY EXPENDITURE | | | | | | | | | |
| C. EXTRAORDINARY EXPENDITURE— | | | | | | | | | |
| I. REPAIRS ... ... ... | | | | | | | | | |
| II. BUILDING IMPROVEMENTS | | | | | | | | | |
| TOTAL EXTRAORDINARY EXPENDITURE ... ... ... | | | | | | | | | |
| | | | | | | | £ | | |

Copies of the Index of Classification and a Synopsis of the "Heads of Charge," together with examples of Classification, will, it is believed, be obtainable in a short time from the Secretary of the Metropolitan Hospital Sunday Fund, Queen Victoria Street, E.C.

